# Relative children’s lipid accumulation with hypertension in Chinese children and adolescents

**DOI:** 10.1186/s12889-021-11868-5

**Published:** 2021-10-19

**Authors:** Zizhe Zhang, Li Zhang, Lili Sun, Bangxuan Wang, Yongting Yuan, Huaiquan Gao, Lianguo Fu

**Affiliations:** Department of child and adolescent health, School of public health, Bengbu Medical College, No. 2600 east sea avenue, Room 207, Bengbu, 233030 Anhui China

**Keywords:** Hypertension, children’s lipid accumulation product, Children and adolescents

## Abstract

**Background:**

This study aimed to develop a novel indicator associated with hypertension in Chinese children and adolescents, the relative children’s lipid accumulation product (RCLAP).

**Methods:**

A cross-sectional study was conducted in 2018. A total of 683 students aged 8–15 years were recruited via a stratified cluster sampling Methods. Anthropometric indexes (waist circumference (WC), Body mass index (BMI), Waist-height ratio (WHtR), logarithm children LAP (LnCLAP), RCLAP per height (RCLAP-H)) were standardized using a z-score method (standardized variables: SWC, SBMI, SWHtR, SLnCLAP, SRCLAP-H). A logistic regression model was performed to evaluate the association of the above indicators with the outcome of hypertension.

**Results:**

The overall prevalence of hypertension was 5.7% (5.5% in boys, 6.0% in girls). SWC ≥ *P*_75_, SBMI ≥ *P*_75_, SWHtR ≥ *P*_75_, SlnCLAP ≥ *P*_75_ and SRCLAP-H ≥ *P*_75_ significantly increased risk of hypertension, with odds ratios (OR) of 2.21 (95% confidence interval (CI): 1.13, 4.30), 2.30 (1.18, 4.49), 2.64 (1.35, 5.14), 4.43 (2.28, 8.61), and 4.49 (2.31, 8.71), respectively.

**Conclusion:**

RCLAP is a novel indicator associated with hypertension in Chinese children and adolescents, and it performs better than WC, BMI, WHtR and children LAP.

## Introduction

Hypertension is the most common risk factor for cardiovascular disease (CVD) morbidity and mortality, and it has been found to be seriously harmful to human health worldwide [1, 2]. Childhood hypertension was once considered a rare disease, but it is now a major public health problem worldwide [3–6]. A meta-analysis of 25 studies showed that the prevalence of hypertension among 943,128 17-year-olds increased steadily from 6.3% in 1995 to 19.2% in 2014 [7]. The Health and Nutrition Survey of Chinese residents reported that the prevalence of childhood hypertension increased significantly from 7.6% in 1993 to 13.8% in 2009 [8].

There is evidence that the root causes of hypertension can be traced back to childhood [9]. Childhood hypertension not only increases the prevalence of adulthood hypertension but also increases the morbidity and mortality from cardiovascular disease [9–13]. The occurrence of hypertension in children and adolescents is asymptomatic, and it is easy to ignore. Therefore, the early detection of elevated blood pressure in children is an important measure to control and prevent adulthood hypertension.

It is well known that many chronic diseases, such as CVD, diabetes and hypertension, are associated with increased body fat and lipid accumulation [14]. Traditionally, the body mass index (BMI), waist-to-height ratio (WHtR) and triglycerides (TG) are the most commonly used indicators to evaluate overall obesity and abdominal obesity in children [15]. However, BMI can only reflect the degree of overweight, but not the fat distribution of the individual. Abdominal fat is more closely related to hypertension than BMI [16, 17]. Although the waist circumference (WC) and WHtR can accurately reflect the degree of abdominal obesity, they cannot distinguish between subcutaneous fat and visceral fat. In addition, a prospective study reported a significant relationship between TG and hypertension [18], but triglycerides were limited to showing adipose tissue accumulation.

In 2005, Kahn et al. [19] put forward the concept of the lipid accumulation product (LAP) for the first time and combined the WC and TG to estimate excessive lipid accumulation in adults. It was found that association between LAP and diabetes was stronger than that between BMI and diabetes in adults [19]. Increasing numbers of studies have shown that LAP has a stronger association with hypertension than other obesity indicators in adults [20–22].

Due to their characteristics of rapid growth and development, LAP cannot be directly used to reflect the lipid accumulation of children and adolescents. Our previous study developed a new indicator, the children lipid accumulation product (children LAP), and confirmed that children LAP was significantly associated with metabolic syndrome and hypertension in children [23, 24]. The children LAP is the product of WC, TG and abdominal skinfold thickness (AST) (children LAP = WC (cm) × AST (mm) × TG (mmol/L) / 100). However, children LAP is limited to reflecting lipid accumulation per unit density of the body. The relative children LAP per unit body height may better reflect the density of lipid accumulation among children and adolescents. This study aimed to evaluate the association of relative children’s lipid accumulation product (RCLAP) with hypertension in Chinese children and adolescents.

## Methods

### Study participants

In this study, a cross-sectional survey among students aged 8–15 years from two nine-year-system schools in Bengbu was conducted in 2018. A total of 683 students (366 boys (53.6%) and 317 girls (46.4%)) were selected by stratified cluster sampling. The study was conducted in accordance with the Declaration of Helsinki (as revised in 2013). The present study was approved by the Medical Ethics Committee of Bengbu Medical College [2015 No. 003] and informed consent was taken from all the participants (or their parents) before the survey.

### Data collection and measurements

#### Anthropometric indices

The medical staff who received standardized training measured the participants’ body weight, height, sitting height (SH), WC, and AST [23]. The participants were required to have an empty stomach, wear light clothes, and be barefoot when measured. Body height and SH were measured using a mechanical height gauge with an accuracy of 0.1 cm. Weight was measured using an electronic weight scale with an accuracy of 0.1 kg. The WC that was the perimeter of the WC at 1 cm above the belly button was measured using nylon tape with an accuracy of 0.1 cm. The AST, which was the skinfold thickness at the junction of the right collarbone midline and belly button horizontal line, was measured using a skinfold thickness gauge with an accuracy of 0.1 mm.

#### Dietary behaviors and physical activity indices

A self-administered food frequency questionnaire was used to investigate dietary behaviors, including breakfast frequency, eggs, milk, fresh vegetables, fruits, nuts, eating out, fried food, carbonated drinks, western fast food, high-energy snacks [23, 24]. The scores of each dietary behavior were as follows: 7 points (7 times / week), 5 points (4 times / week), 2 points (1 time / week), 0.5 points (2 times / month), 0.25 points (1 time / month) and 0 points (never). Eating behaviors were divided into healthy and unhealthy dietary behaviors (healthy dietary behaviors including breakfast, eggs, milk, fresh vegetables, fruits, nuts; unhealthy dietary behaviors including eating out, fried foods, carbonated drinks, Western fast food, high-energy snacks). The total scores of healthy eating behaviors and unhealthy eating behaviors were calculated. Cronbach’s alpha coefficients of healthy dietary behaviors and unhealthy dietary behaviors scores were 0.705 and 0.700, respectively. According to the 75th percentile (*P*_75_) of participants with healthy eating behaviors were divided into ≥ *P*_75_ group and < *P*_75_ group. According to the 75th percentile (*P*_75_) of participants with unhealthy eating behaviors were divided into ≥ *P*_75_ group and < *P*_75_ group. The Chinese Children’s Leisure Activities Study Survey questionnaire (CLASS-C) [25] was used to investigate the time of moderate to severe physical activity and sedentary activity. The time of moderate or strenuous exercise was divided into two grades: ≥ 60 min and < 60 min [26]. The time of sedentary activity was divided into two levels: ≥ 120 min and < 120 min [27].

#### Blood lipid measurements

Three milliliters of fasting venous blood were collected from each student at the physical examination center of a grade III, class A hospital. The collected blood samples were centrifuged within 2 h and TG (mmol/L) was detected by an enzymatic method using an automated biochemical analyzer.

#### Blood pressure measurement

After having a rest for at least 10 min, appropriate cuffs were selected according to age and arm circumference of the subjects, and the systolic blood pressure (SBP) and diastolic blood pressure (DBP) on the right arm were measured using a mercury sphygmomanometer. SBP was defined as the onset of the Korotkoff sound (K1), and DBP was defined as the fifth Korotkoff sound (K5). The blood pressure was measured at an interval of 2 min, and the average of the two measurements was recorded as the final blood pressure value.

#### Definition of hypertension

In this study, hypertension was diagnosed according to “Reference of screening for elevated blood pressure among Chinese children and adolescents aged 7–18 years” (Health industry standard of the People’s Republic of China: WS/T 610–2018) [28].

#### Calculation of derivative indicators

BMI = weight (kg) / height^2^ (m^2^); WHtR = WC (cm) / height (cm); Children LAP = WC (cm) × AST (mm) × TG (mmol/L) / 100. Relative children’s lipid accumulation product per height (RCLAP-H) = WC (cm) × AST (mm) × TG (mmol/L) / height (cm).

### Statistical analysis

The sample size was determined according to the cross-sectional design of the current situation. The calculation formula was $n=\frac{\mu_{\alpha /2}^2p\left(1-p\right)}{\delta^2}$ (where α = 0.05, δ = 0.03, and *p* is the prevalence of hypertension). A study showed that the prevalence of hypertension in children aged 6–13 years in China was 18.4% [29]. According to the formula of sample size, the sample size should be 641(641 = 1.96 × 1.96 × 0.184 × (1–0.184)/0.03/0.03), however, the 5% of sample size needed to be increased for sampling error. Therefore, the minimum sample size was 673 (673 = 641 × (1 + 1.05)). In fact, the effective sample size was 683. SPSS 23.0 software was used for statistical analysis. The measurement and counting data are described by average with 95% confidence interval (CI) and ratio or proportion, respectively. Logarithm Children LAP (LnCLAP), RCLAP-H, height, weight, SH, WC, WHtR, BMI, AST and TG were standardized by sex and age using z-scores (standardized variables: SLnCLAP, SRCLAP-H, Sheight, Sweight, SSH, SWC, SWHtR, SBMI, SAST and STG). T-tests was used to compare the differences in above standardized indices between children with hypertension and non-hypertension. Chi-square tests were used to analyze associations between above standardized indices and hypertension. Furthermore, after adjusting for moderate-to-vigorous physical activity time, sedentary activity time, healthy dietary behaviors and risky dietary behaviors, the logistic regression models were used to analyse the relations between anthropometric indexes and hypertension.

## Results

The overall prevalence of hypertension was 5.7% (5.5% for boys and 6.0% for girls). From Table 1, we see that SSH, Sweight, SWC, SAST, STG, SBMI, SWHtR, SlnCLAP and SRCLAP-H were significantly lower in non-hypertensive children than they were in hypertensive children (*P* < 0.05). The mean difference in standardized units of two groups with 95%CI of Sheight, SSH, Sweight, SWC, SAST, STG, SBMI, SWHtR, SlnCLAP and SRCLAP-H were − 0.17 (− 0.49, 0.15), − 0.33 (− 0.65, − 0.01), − 0.32 (− 0.64, − 0.01), − 0.59 (− 0.98, − 0.2), − 0.69 (− 1.00, − 0.37), − 0.43 (− 0.85, − 0.02), − 0.32 (− 0.64, 0.00), − 0.59 (− 1.02, − 0.15), − 0.64 (− 0.96, − 0.33) and − 0.83 (− 1.29, − 0.37), respectively.

**Table 1:** Differences in standardized anthropometric indexes in children with hypertension and non-hypertension. (*n* = 683)

Variables	Non-hypertension Group (*n* = 644) *Mean(95%CI)*	Hypertension Group (*n* = 39) *Mean(95%CI)*	Mean Difference *Mean(95%CI)*	*P*-value
Sheight	−0.01 (− 0.09, 0.07)	0.16 (− 0.13, 0.45)	−0.17 (− 0.49, 0.15)	0.300
SSH	−0.02 (− 0.10, 0.06)	0.31 (0.00, 0.62)	−0.33 (− 0.65, − 0.01)	0.044
Sweight	−0.02 (− 0.10, 0.06)	0.31 (0.02, 0.59)	−0.32 (− 0.64, − 0.01)	0.047
SWC	−0.03 (− 0.11, 0.04)	0.56 (0.17, 0.94)	−0.59 (− 0.98, − 0.2)	0.004
SAST	−0.04 (− 0.11, 0.04)	0.65 (0.30, 1.00)	−0.69 (− 1.00, − 0.37)	< 0.001
STG	− 0.02 (− 0.10, 0.05)	0.41 (0.00, 0.82)	−0.43 (− 0.85, − 0.02)	0.043
SBMI	−0.02 (− 0.09, 0.06)	0.30 (− 0.04, 0.65)	−0.32 (− 0.64, 0.00)	0.048
SWHtR	−0.03 (− 0.11, 0.04)	0.55 (0.13, 0.98)	−0.59 (− 1.02, − 0.15)	0.009
SLnCLAP	− 0.04 (− 0.11, 0.04)	0.61 (0.26, 0.95)	−0.64 (− 0.96, − 0.33)	< 0.001
SRCLAP-H	−0.05 (− 0.12, 0.03)	0.79 (0.33, 1.24)	−0.83 (− 1.29, − 0.37)	0.001

*Abbreviations*: *Sheight* Standardized height, *SSH* Standardized siting height, *Sweight* Standardized weight, *SWC* Standardized waist circumference, *SAST* Standardized abdominal skinfold thickness, *STG* Standardized triacylglycerol, *SBMI* Standardized body mass index, *SWHtR* Standardized waist-height ratio, *SlnCLAP* Standardized logarithmic children’s lipid accumulation product, *SRCLAP-H* Standardized relative children’s lipid accumulation product per height

As shown in Table 2. The moderate-to-vigorous physical activity time, sedentary activity time, healthy dietary behaviors and unhealthy dietary behaviors were not significantly related to hypertension in these children (*P* > 0.05). There was no significant difference in Sheight and SSH between hypertensive and non-hypertensive children (*P* > 0.05). The results of chi-square test showed that Sweight, SWC, SAST, STG, SBMI, SWHtR, SlnCLAP and SRCLAP-H were significantly associated with hypertension in sample children (*P* < 0.05).

**Table 2:** Standardized differences in anthropmetric indexes, dietary behaviors and physical activity in children with hypertension and non-hypertension. (*n* = 683)

Variables	Non-hypertension Group (*n* = 644) *n(%)*	Hypertension Group (*n* = 39) *n(%)*	*x*^2^	*P-*value	*OR (95%CI)*
Sex			0.09	0.766	0.91 (0.48, 1.73)
Girls	298(46.3)	19(48.7)			
Boys	346(53.7)	20(51.3)			
Ages			3.14	0.077	1.78 (0.93, 3.41)
8~	405(62.9)	19(48.7)			
12 ~ 15	239(37.1)	20(51.3)			
Sheight			0.05	0.820	0.92 (0.43, 1.97)
<*P*_75_	485(75.3)	30(76.9)			
≥*P*_75_	159(24.7)	9(23.1)			
SSH			1.58	0.210	1.55 (0.78, 3.09)
<*P*_75_	487(75.6)	26(66.7)			
≥*P*_75_	157(24.4)	13(33.3)			
Sweight			4.18	0.041	1.99 (1.02, 3.89)
<*P*_75_	490(76.1)	24(61.5)			
≥*P*_75_	154(23.9)	15(38.5)			
SWC			5.51	0.019	2.18 (1.12, 4.22)
<*P*_75_	488(75.8)	23(59.0)			
≥*P*_75_	156(24.2)	16(41.0)			
SAST			15.64	< 0.001	3.50 (1.82, 6.73)
<*P*_75_	495(76.9)	19(48.7)			
≥*P*_75_	149(23.1)	20(51.3)			
STG			7.44	0.006	2.44 (1.26, 4.71)
<*P*_75_	489(75.9)	22(56.4)			
≥*P*_75_	155(24.1)	17(43.6)			
SBMI			5.63	0.018	2.20 (1.13, 4.26)
<*P*_75_	489(75.9)	23(59.0)			
≥*P*_75_	155(24.1)	16(41.0)			
SWHtR			7.74	0.005	2.48 (1.28, 4.79)
<*P*_75_	491(76.2)	22(56.4)			
≥*P*_75_	153(23.8)	17(43.6)			
SLnCLAP			21.98	< 0.001	4.34 (2.24, 8.38)
<*P*_75_	496(77)	17(43.6)			
≥*P*_75_	148(23)	22(56.4)			
SRCLAP~H			22.57	< 0.001	4.41 (2.28, 8.53)
<*P*_75_	498(77.3)	17(43.6)			
≥*P*_75_	146(22.7)	22(56.4)			
Moderate-to-vigorous physical activity time			1.55	0.213	0.65 (0.33, 1.28)
< 60 min	347(53.9)	25(64.1)			
≥60 min	297(46.1)	14(35.9)			
Sedentary activity time			2.05	0.153	1.63 (0.83, 3.19)
< 120 min	307(47.7)	14(35.9)			
≥120 min	337(52.3)	25(64.1)			
Healthy dietary behaviors			1.03	0.311	0.65 (0.28, 1.50)
<*P*_75_	482(74.8)	32(82.1)			
≥*P*_75_	162(25.2)	7(17.9)			
Risk dietary behaviors			0.29	0.590	1.22 (0.59, 2.50)
<*P*_75_	487(75.6)	28(71.8)			
≥*P*_75_	157(24.4)	11(28.2)			

*Abbreviations*: *Sheight* Standardized height, *SSH* standardized siting height, *Sweight* Standardized weight, *SWC* Standardized waist circumference, *SAST* Standardized abdominal skinfold thickness, *STG* Standardized triacylglycerol, *SBMI* Standardized body mass index, *SWHtR* Standardized waist-height ratio, *SlnCLAP* Standardized logarithmic children’s lipid accumulation product, *SRCLAP-H* Standardized relative children’s lipid accumulation product per height

As shown in Table 3, Logistic models of the relationship between anthropometric indices and hypertension were setted up. After adjusting for moderate-to-vigorous physical activity time, sedentary activity time, healthy dietary behaviors and risky dietary behaviors, then factors Sweight, SWC, SAST, STG, SBMI, SWHtR, SlnCLAP and SRCLAP-H were separately entered into the logistic regression models as covariates. The results of the logistic regression models showed that the z-sores ≥*P*_75_ groups of Sweight, SWC, SAST, STG, SBMI, SWHtR, SlnCLAP and SRCLAP-H were significantly associated with an increased risk of hypertension in children (*P* < 0.05), with odds ratios (OR) of 1.99 (95%CI: 1.02, 3.91), 2.21 (1.13, 4.30), 3.61 (1.86, 6.99), 2.43 (1.25, 4.71), 2.30 (1.18, 4.49), 2.64 (1.35, 5.14), 4.43 (2.28, 8.61), and 4.49 (2.31, 8.71), respectively. In addition, Sweight, SWC, SAST, STG, SBMI, SWHtR, SlnCLAP and SRCLAP-H as independent variables were put into logistic model, after adjusting for moderate-to-vigorous physical activity time, sedentary activity time, healthy dietary behaviors and risky dietary behaviors, the result of stepwise logistic model (forward conditional method) showed only SRCLAP-H ≥ *P*_75_ significantly increased risk of hypertension (OR (95%CI) = 4.49 (2.31, 8.71)).

**Table 3:** The associations between different groups of anthropometric indexes and hypertension using logistic regressions

Variables	*OR* *(95%CI)*	*P*-value
Sweight		
≥ *P*_75_	1.99 (1.02, 3.91)	0.045
SWC		
≥ *P*_75_	2.21 (1.13, 4.30)	0.020
SAST		
≥ *P*_75_	3.61 (1.86, 6.99)	< 0.001
STG		
≥ *P*_75_	2.43 (1.25, 4.71)	0.009
SBMI		
≥ *P*_75_	2.30 (1.18, 4.49)	0.015
SWHtR		
≥ *P*_75_	2.64 (1.35, 5.14)	0.004
SlnCLAP		
≥ *P*_75_	4.43 (2.28, 8.61)	< 0.001
SRCLAP-H		
≥ *P*_75_	4.49 (2.31, 8.71)	< 0.001

*Abbreviations*: *Sweight* Standardized weight, *SWC* Standardized waist circumference, *SAST* Standardized abdominal skinfold thickness, *STG* Standardized triacylglycerol, *SBMI* Standardized body mass index, *SWHtR* Standardized waist-height ratio, *SlnCLAP* Standardized logarithmic children’s lipid accumulation product, *SRCLAP-H* Standardized relative children’s lipid accumulation product per height

## Discussion

The prevalence of childhood hypertension has been increasing worldwide [30]. The current research showed that the overall prevalence of childhood hypertension was 5.7% (5.5% for boys and 6.0% for girls), which was generally lower than that reported at the national level. According to a national survey, the prevalence of hypertension among 35,657 Chinese teenagers aged 17 years old was 12.4% (12.4% in boys and 12.3% in girls) [31]. Reports from the Chinese National Survey on Students’ Constitution and Health (CNSSCH) showed that the prevalence of elevated blood pressure among children in 2010 was 16.1% for boys and 12.9% for girls [32]. The reasons for the discrepancies may be regional and population differences in children’s blood pressure distribution; alternatively, the diagnostic criteria, children’s ages, and sample size in this study were different from other studies. A meta-analysis also found that the overall prevalence of hypertension among preschool children in China was only 5% (95% CI: 4%, 9%) [33]. In addition, this study found that there were no significant differences in moderate to strenuous physical activity time, sedentary activity time, healthy eating behaviors and unhealthy eating behaviors between children with hypertension and non-hypertensive children. Qu et al. [34] reported the consistent findings that physical activity intensity and dietary behavior management had nothing to do with the occurrence of hypertension in children. However, some studies also reported that an increase of physical activity intensity and time decreased the incidence of hypertension and that an unhealthy diet increased the risk of hypertension [35–37].

There is growing evidence that an increase of hypertension in children is closely related to weight gain [38]. BMI is an internationally recognized index for screening obesity. Previous studies showed that BMI was also associated with hypertension in children and adolescents [39–41]. In this study, it was found that the body weight and BMI in hypertensive children were significantly higher than those in non-hypertensive children. Although BMI provides a simple and convenient measure of obesity, it does not measure the distribution of body fat [42]. The mechanism of childhood obesity leading to hypertension is very complex [43]. It is worth noting that the functions of different adipose tissues are very different [44]. Some studies have pointed out that abdominal fat is more closely related to hypertension in children than BMI [45].

WC and WHtR can accurately reflect the degree of abdominal obesity. One study showed that an increase of WC in children with a normal weight was associated with an increase in blood pressure [46]. A study by Cruz et al. [47] showed that waistline was a good predictor of elevated blood pressure in children aged 8 to 10 years old. This study also found that the WC in hypertensive children was significantly higher than that in non-hypertensive children, and SWC ≥ *P*_75_ significantly increased the risk of hypertension (OR (95% CI): 2.21 (1.13, 4.30). Lo K et al. [48] reported that the WC was more closely associated with cardiac metabolism and hypertension in children compared with BMI, WC, and WHtR. Some researchers believe that WHtR is an important indicator of obesity and is more likely to predict cardiovascular risk than BMI [49]. In this study, we also found that there was a significant correlation between WHtR and hypertension in children, with an OR (95% CI) of 2.64 (1.35, 5.14). Previous studies [23, 24] reported that children LAP was significant associated with hypertension or metabolic syndrome in Chinese children and adolescents as compared with BMI and WHtR.

This study found that BMI, WC, WHtR, and children LAP were significantly associated with hypertension in children, which is consistent with previous studies. However, BMI is limited to distinguishing the ratio of body fat to muscle tissue and not the distribution of body fat [19]. WC and WHtR can only show the accumulation of fat in the blood circulation [50]. Children LAP only reflects the accumulation of adipose tissue and lipids in the blood circulation, while neglecting the accumulation of lipids per unit density of the human body.

The present study showed that SRCLAP-H in hypertensive children was significantly higher than it was in non-hypertensive children; SRCLAP-H ≥ *P*_75_ was significantly associated with an increased risk of hypertension (OR (95% CI) was 4.49 (2.31, 8.71), which were higher than those of WC, BMI, WHtR, and children LAP, respectively). Therefore, compared with WC, BMI, WHtR, and children LAP, RCLAP-H was more strongly associated with hypertension on the basis of the same critical values. In addition, after adjusting for physical activity time and dietary behavior factors, the stepwise logistic model (forward conditional method) showed SRCLAP-H significantly increased risk of hypertension, while SWC, SBMI, SWHtR and SlnCLAP did not, which means RCLAP-H may be more strongly associated with hypertension than other indicators. We speculate that RCLAP may be more suitable for differential diagnosis of hypertension in children and adolescents. RCLAP-H reflects the accumulation of lipids per unit height. Children with the same children LAP but with a shorter height may have a higher risk of developing high blood pressure.

However, this study has some limitations. First, this was a cross-sectional study. Therefore, the causal relationship between RCLAP and hypertension cannot be inferred. Second, there is no biological or rationale for the calculation of RCLAP. In addition, this study used a self-administered food frequency questionnaire which has not been validated elsewhere. Blood pressure was measured only twice in this study, which could generate misclassification bias. 3 measurements and taking the average of the last 2 measurements would be ideal. Finally, we only studied Chinese children and adolescents, and the generalizability of our findings to other countries is limited and needs additional data for confirmation.

## Conclusion

In conclusion, it is crucial to assess visceral fat accumulation in a convenient and cheap way for the prevention of early CVD in children and adolescents. Compared with traditional obesity indicators such as BMI, WC and WHtR, RCLAP-H can better reflect the density of lipid accumulation per unit height among children and adolescents, rather than simple high body weight or abdominal fat thickness. Our study suggests that the relative children’s lipid accumulation product (RCLAP-H) is associated with hypertension. As traditional assessment methods of visceral fat evaluation are not available in daily clinical application, RCLAP can be extensively used in epidemiological studies and some large-scale clinical trials. Due to the sample size in our study is limited, RCLAP also requires extensive population data screening to determine criteria for wider guidance in clinical practice.

## Data Availability

The datasets used and/or analysed during the current study are available from the corresponding author on reasonable request.

## Abbreviations

SH: Standardized height
SSH: Standardized sitting height
Sweight: Standardized weight
WC: Waist circumference
SWC: Standardized waist circumference
AST: Abdominal skinfold thickness
SAST: Standardized abdominal skinfold thickness
TG: Triglycerides
STG: Standardized triglycerides
BMI: Body mass index
SBMI: Standardized body mass index
WHtR: Waist-height ratio
SWHtR: Standardized Waist-height ratio
CVD: Cardiovascular disease
SBP: Systolic blood pressure
DBP: Diastolic blood pressure
LAP: Lipid accumulation product
Children LAP: Children’s lipid accumulation product
SlnCLAP: Standardized logarithmic children’s lipid accumulation product
RCLAP-H: Relative children’s lipid accumulation product per height
SRCLAP-H: Standardized relative children’s lipid accumulation product per height
